# Infantile Cerebellar‐Retinal Degeneration Associated With Novel *ACO2* Variants: Clinical Features and Insights From a *Drosophila* Model

**DOI:** 10.1111/cge.14745

**Published:** 2025-04-10

**Authors:** Edgar Buhl, Suchika Garg, Marie Monaghan, Amy Preston, Marcus Likeman, Julianne Dare, Julie Evans, Lucie S. Taylor, Ian Berry, Kathryn Urankar, Paul G. D. Spry, Cathy Williams, Robert W. Taylor, Charlotte L. Alston, James J. L. Hodge, Anirban Majumdar

**Affiliations:** ^1^ School of Physiology, Pharmacology and Neuroscience University of Bristol Bristol UK; ^2^ Department of Paediatric Neurology University Hospitals Bristol NHS Foundation Trust Bristol UK; ^3^ Department of Paediatric Neuroradiology University Hospitals Bristol NHS Foundation Trust Bristol UK; ^4^ Department of Community Paediatrics, Community Children's Health Partnership Kingswood Locality Hub Bristol UK; ^5^ Bristol Genetics Laboratory North Bristol NHS Trust Bristol UK; ^6^ Mitochondrial Research Group, Translational and Clinical Research Institute, Faculty of Medical Sciences Newcastle University Newcastle Upon Tyne UK; ^7^ NHS Highly Specialised Service for Rare Mitochondrial Disorders Newcastle Upon Tyne Hospitals NHS Foundation Trust Newcastle Upon Tyne UK; ^8^ Department of Neuropathology North Bristol Hospital NHS Foundation Trust Bristol UK; ^9^ Department of Paediatric Ophthalmology University Hospitals Bristol NHS Foundation Trust Bristol UK

**Keywords:** *ACO2*, *Drosophila melanogaster*, ERG, infantile cerebellar‐retinal degeneration, locomotion, *mAcon1*, MRI, optic atrophy, sleep

## Abstract

Infantile Cerebellar‐Retinal Degeneration (ICRD) is an autosomal recessive neuro‐disability associated with hypotonia, seizures, optic atrophy, and retinal degeneration. Recessive variants of the mitochondrial aconitase gene (*ACO2*) are a known cause of ICRD. Here, we present a paediatric male patient with ICRD, where whole genome sequencing of the family trio revealed segregating heterozygous variants of unknown significance in *ACO2*. At 4 months, he displayed generalised hypotonia, and by 6 years, visual electrophysiology indicated bilateral optic atrophy. Magnetic Resonance Imaging (MRI) at age seven confirmed optic nerve and cerebellar atrophy, and together with symptoms of developmental delay, align with ICRD. We established a *Drosophila* animal model to explore the impact of *ACO2* loss‐ and gain‐of‐function. Manipulating the fly ortholog, *mAcon1*, through pan‐neuronal knock‐down or over‐expression negatively affected longevity, locomotion, activity, whilst disrupting sleep and circadian rhythms. Mis‐expression of *mAcon1* in the eye led to impaired visual synaptic transmission and neurodegeneration. These experiments mirrored certain aspects of the human disease, providing a foundation for understanding its biological processes and pathogenic mechanisms, and offering insights into potential targets to screen for future treatments or preventive measures for *ACO2*‐related ICRD.

## Graphical abstract

Our Translational Loop integrates patient genetic data with *Drosophila* models to study disease mechanisms. We identified *ACO2* variants in a patient linked to ICRD and show that our animal model mirrors key aspects of the disease. These insights help pinpoint therapeutic targets, advancing research toward treatments for rare genetic disorders.

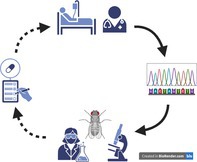

## 1 Introduction

Infantile Cerebellar Retinal Degeneration (ICRD) OMIM #614559 is an autosomal recessive disorder caused by homozygous or compound heterozygous variants in the human aconitase 2 (*ACO2)* gene (located on chromosome 22q13.2). The condition has a prevalence of < 1:1 000 000, with less than 30 patients reported worldwide [1]. Symptoms classically develop by 6 months of age and include, but are not limited to, truncal hypotonia, athetosis (constant writhing movements), seizures, optic atrophy and retinal degeneration; while other patients have milder symptoms of ataxia, developmental delay, and behavioural abnormalities [2].

Detailed biochemical analysis by Abela and colleagues [3] elucidated the role of *ACO2* in intracellular signalling pathways. *ACO2* is a cluster protein, an isoform of aconitase, responsible for the reversible isomerisation of the first step of the Kreb's cycle, namely metabolising citric acid to isocitric acid in the mitochondria. *ACO2* deficiency leads to reduced levels of alpha‐ketoglutarate and increased glutamate levels; both thought to lead to neurotoxicity in patients with pathogenic *ACO2* variants. Previously, only recessive *ACO2* variants had been identified in association with human pathology, but a recent study has found dominant *ACO2* variants cause a similar clinical presentation, with onset later in childhood [4]. Both monoallelic (dominant) and biallelic (recessive) *ACO2* variants are predicted to impair substrate binding and lower enzyme activity [5]. Within the United Kingdom NHS Genomic Medicine Service (GMS), *ACO2* is interrogated as part of 12 distinct virtual panels, eight of which have diagnostic grade ‘green status’ as per the Genomics England PanelApp [6].

The fly ortholog of human *ACO2* is the mitochondrial aconitase 1 gene (*mAcon1*) that has a high DIOPT (DRSC Integrative Ortholog Prediction Tool v9.1) score of 14/16 (71% amino acid identity; https://flybase.org/reports/FBgn0010100 [7]. In humans, *ACO2* is expressed ubiquitously and is implicated in ICRD and optic atrophy [8]. In flies, *mAcon1* is also expressed ubiquitously, with knock‐out mutants being embryonically lethal.

We describe the presentation and six‐year follow‐up of a patient with causative biallelic *ACO2* variants and describe the investigative work‐up as well as the dynamic changes seen on MRI imaging correlating to clinical function. Furthermore, we employed the fruit fly, *Drosophila melanogaster*, as an animal model to investigate potential biological mechanisms and pathogenic pathways underlying the clinical phenotype [9]. For this, we utilised the Gal4/UAS system [10], a binary yeast transcription enhancer system allowing tissue‐specific expression of transgenes, in this case throughout the whole nervous system (using *elav‐Gal4*) or in the eyes (*GMR‐Gal4*), to specifically downregulate (via RNA interference, *mAcon1* ^*RNAi*^) or upregulate (by over‐expression, *mAcon1* ^*OX*^) expression of *mAcon1* to determine whether the *Drosophila* orthologue recapitulates the features of human *ACO2* and elucidate underlying disease mechanisms.

## 2 Materials and Methods

Detailed procedures can be found in Supplementary Methods S1.

## 3 Results

### 3.1 Case Report and Clinical Features

**FIGURE 1:**
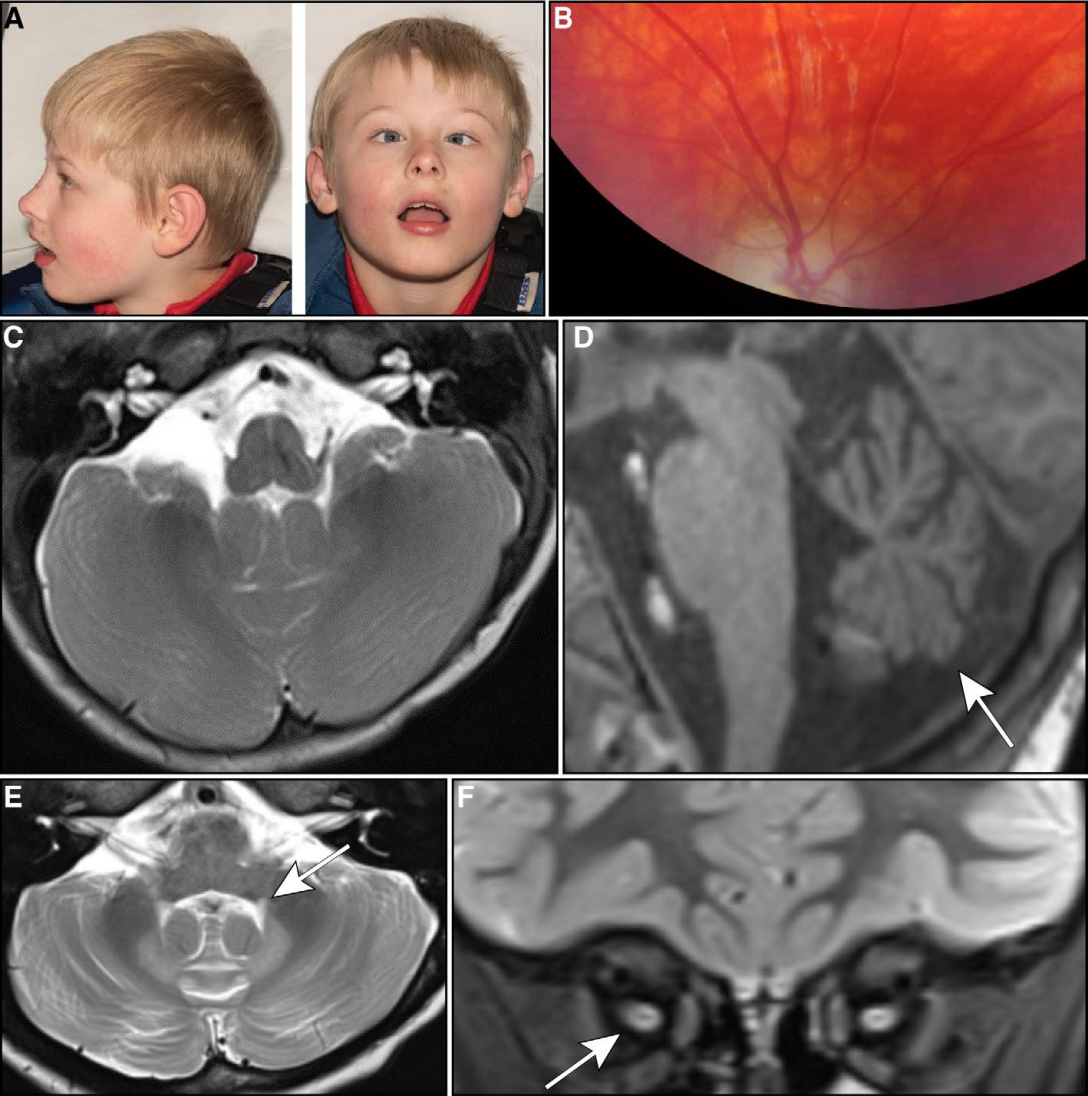
Clinical features and neurological manifestations in MRI imaging. (A) Clinical presentation of bilateral large angle esotropia (squint), hypotelorism (decreased distance between the orbits), plagiocephaly (asymmetrical flattening of the head) and facial telangiectasias (dilation of capillaries) observed in the patient. (B) Photo of Right retina showed optic atrophy in the form of severe optic disc pallor. (C–E) Sequential MRI images illustrating the progression of neurological changes: (C) a normal volume of cerebellar hemispheres in an axial T2 sequence at age 1, (D) development of cerebellar vermian hypoplasia (arrow) at age 7 in a sagittal T1 sequence, and (E) symmetrical volume loss of the cerebellar hemispheres accompanied by high intensity in the dentate nuclei in an axial T2 sequence. A subtle finding of minimal prominence of the central tegmental tract (arrow) was also noted in the MRI analysis. (F) Coronal STIR sequence, revealing slender intra‐orbital optic nerves with more severe atrophy on the left side (arrow).

The patient was a male infant born at 42 weeks gestation by vaginal delivery weighing 3.15 kg (25th–50th percentile). His birth was uncomplicated, with no need for resuscitation or feeding support. He was admitted to the neonatal unit on the first day of life for transient hypothermia. By 4 months, poor head control was noted, and he was observed to be floppy. At 12 months, motor delay was identified, as the patient was not able to roll or lift his head. At 18 months, he was referred to a paediatric neurology clinic, where examination showed poor central tone, inability to sit or bear weight through his legs, and weight and head circumference were on the ninth centile. He demonstrated age‐appropriate fine motor and social skills and had normal reflexes. Other distinctive features included close‐set eyes, plagiocephaly (asymmetrical flattening of the head) and he had flexible equinovalgus (foot deformity causing the foot to bend down and out; Figure 1A).

At 12 months, he was referred to ophthalmologists for a bilateral large‐angle squint with restricted abduction associated with nystagmus on attempted abduction. He wears glasses for refractive errors, and his hearing was normal. His family history was unremarkable, with no history of neurological disease, and his older sister was developing normally. His mother had a balanced translocation between chromosomes 1 and 16 (46, XX, t(1;16) (p10;q10)/46, XX) identified during chromosomal testing for her own mother's amniocentesis findings, but this was considered benign and unrelated to the patient's presentation.

From the age of 18 months, he received regular physiotherapy and speech and language therapy sessions. He continues to experience motor and speech difficulties, with expressive and receptive language delay and a limited vocabulary of simple words. His chewing, swallowing and diet remained normal. At 4 years, he started having blank episodes and night terrors which self‐resolved. From the age of five, he developed bilateral fixed hip dislocations and now wears a full‐body Lycra suit to help prevent scoliosis. Further examination findings reveal arachnodactyly with overlapping of the second and third toes of the right foot, which was also present in his father. He has good hand function, mobilising and turning his wheelchair independently, with a right‐hand preference. By seven, he became wheelchair‐dependent when outdoors, requiring the use of a fully supported stander when indoors. Currently, 10 years old, he can sit upright with support, mobilises with a manual wheelchair, and can commando crawl. There are currently no cardiological or respiratory concerns; an echocardiogram was normal. Neurologically, he remains hypotonic, has present reflexes, good upper body strength, but no anti‐gravity movements of the lower limbs.

### 3.2 Investigations

#### 3.2.1 Visual Electrophysiology and Electroretinography

To investigate visual function and assess for visual impairment, a clinical examination of the fundi of the retina was conducted, which showed pallor, confirming optic atrophy (Figure 1B). Visual Evoked Potentials (VEP) and an Electroretinogram (ERG) were carried out at 6 years of age (Supplementary Table S2). Retinal cone ERG responses showed evidence of cone system function in both eyes, with a and b component responses exhibiting normal latency and amplitude parameters. However, analysis of cortical responses showed no repeatable right flash VEP responses despite simultaneous cone ERG responses, and a significantly reduced P2 component amplitude in the left flash VEP response. These findings suggest bilateral optic atrophy and visual impairment, despite cone system function.

#### 3.2.2 Imaging

An initial brain MRI at 1 year old showed a normal feature of slight prominences of extra axial cerebrospinal fluid (CSF) spaces over the anterior convexities bilaterally (Figure 1C), with no structural abnormalities and appropriate myelination for age. Over time, MRI changes developed, prompting a repeat MRI scan of his brain and orbits at the age of seven, considering the genetic findings from the 100 000‐genome project and persistent clinical ataxia. This scan revealed cerebellar hemisphere and vermis volume loss, high intensity signal in the dentate nuclei (Figure 1D,E), and atrophy of bilateral optic nerves, more severe on the left (Figure 1F). Compared to the initial scan, the prominence of the convexity subarachnoid spaces had resolved, and myelination remained normal. No abnormalities were identified within the cerebral hemispheres, and the brainstem appeared normal. The abnormalities seen in the brain MRI scan were consistent with previously reported cases of pathogenic ACO2‐related pathology [1].

An x‐ray of his pelvis at 5 years showed bilateral coxa valga (deformity of the neck of the femur). Femoral capital epiphyses (growth plate of the head of the femur) were symmetrically ossified, and acetabula were dysplastic and shallow. There was also moderate subluxation (partial dislocation) of the femoral heads (migration index 58% on right, and 69% on left).

#### 3.2.3 Blood and Cerebrospinal Fluid Tests

Metabolic blood tests, including thyroid function tests, plasma creatine kinase, plasma amino acids, ferritin, and urine organic acids, carried out at presentation were all normal. CSF tests, including glucose, lactate, viral PCR (polymerase chain reaction), and neurotransmitter metabolites, did not identify any detectable abnormalities.

#### 3.2.4 Muscle Biopsy

**FIGURE 2:**
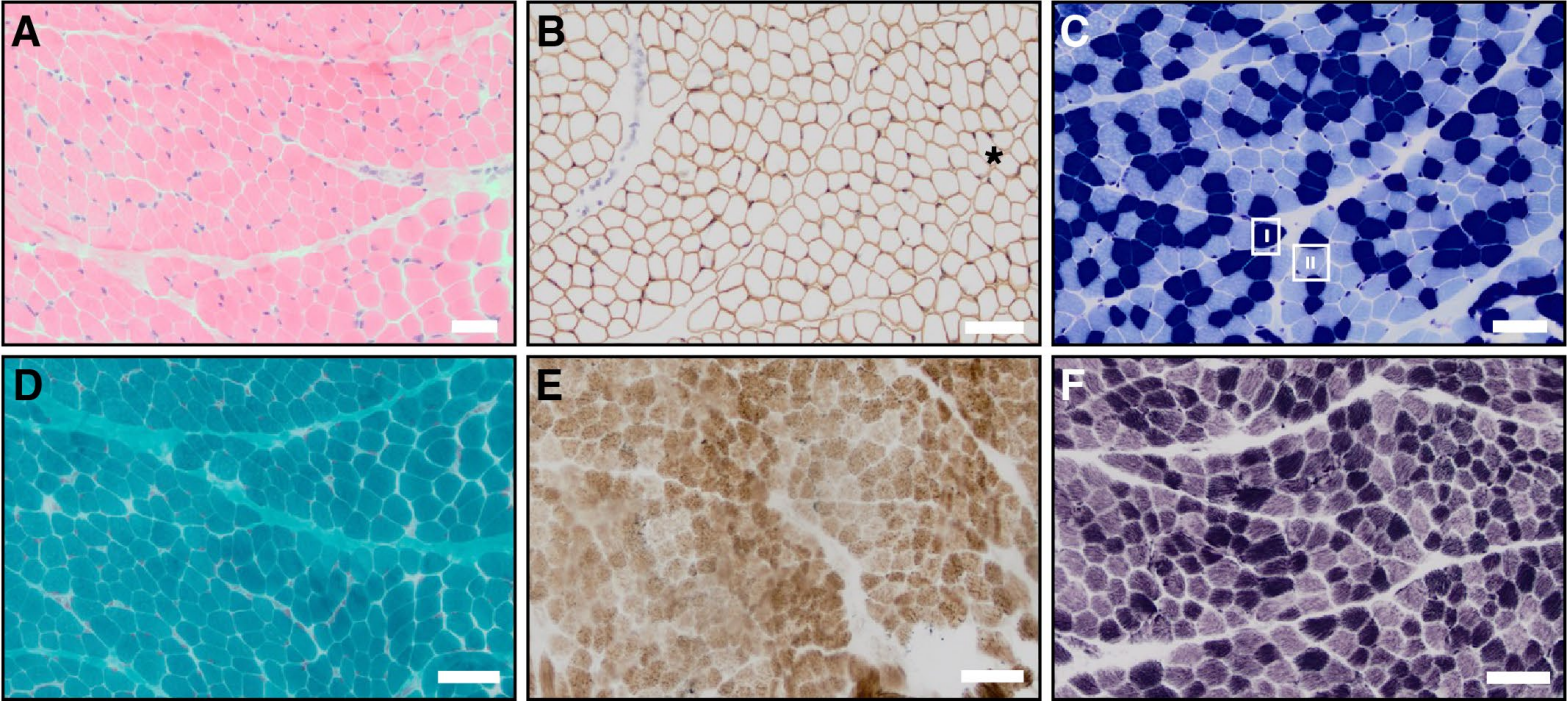
Right thigh (quadriceps) muscle biopsy obtained at age 3. (A) HH&E stain demonstrating mild variation in myofibre diameters, with the overall morphology revealing slight hypotrophy in some fibres. There was also a subtle but notable increase in endomysial connective tissue, suggestive of early, mild myopathic changes. However, no inflammatory infiltrates, necrotic fibres, or regenerating fibres were visualised, indicating an absence of active myonecrosis. (B) Spectrin immunostain highlighting the variation in myofiber diameters (*), aiding in the differentiation of smaller and larger fibres. Spectrin, a cytoskeletal protein, outlines the sarcolemmal membranes in intact fibres. As this was well preserved in all myofibres, it confirms an absence of muscle fibre necrosis. This stain also helps highlight an absence of fibre splitting and a lack of central nucleation in this case, further supporting the presence of only mild non‐specific myopathic features. (C) MATPase stain distinguishes fibre types, with type I (slow oxidative) fibres appearing dark blue and type II (fast glycolytic) fibres light blue. In this case, the MATPase stain revealed type I fibre hypotrophy, contributing to the mild non‐specific myopathic pattern. (D) Gomori Trichrome stain displaying a normal staining pattern with no evidence of ragged red fibres, nemaline rods, or other inclusions, further excluding specific congenital myopathies or mitochondrial disorders. (E) Combined COX‐SDH reaction revealing a normal staining pattern of myofibres and normal oxidative phosphorylation enzyme activity. The absence of COX‐deficient, SDH‐positive fibres suggests that there are no detectable mitochondrial abnormalities within the muscle, consistent with the lack of metabolic dysfunction observed in laboratory investigations. (F) NADH staining showing normal myofibrillar architecture within muscle fibres. There were no visible structural abnormalities such as cores, targets or disruptions in myofibrils, supporting the non‐specific nature of the myopathic features observed. Scale bars, 50 μm.

Given the initial clinical findings of hypotonia, the possibility of a congenital myopathy or metabolic myopathy was raised. Consequently, a skeletal muscle biopsy from the right quadriceps muscle was performed at 3 years of age, revealing mild non‐specific myopathic features, including mild variation in myofiber diameters with hypotrophy of type 1 (slow) fibres. Histochemical stains showed no structural abnormalities characteristic of common congenital myopathies (Figure 2A–C). Analysis with Gomori trichrome, SDH (Succinate Dehydrogenase; aka Complex II) and COX‐SDH (Cytochrome *c* Oxidase‐Succinate Dehydrogenase; corresponding to Complex IV and Complex II, respectively, of the mitochondrial respiratory chain) was performed. Loss of COX/Complex IV activity is typically identified by retention of SDH/Complex II staining of muscle fibres. The stains revealed no ragged red fibres, ragged blue fibres, or COX‐deficient fibres to suggest an underlying mitochondrial myopathy (Figure 2D–F). No rods were observed excluding Nemaline myopathy. Additionally, there were no histological features of congenital muscular dystrophy, and immunohistochemical staining for dystrophin‐associated proteins was normal. Electron microscopy showed no significant ultrastructural abnormalities, including mitochondrial inclusions.

Biochemical assessment of mitochondrial respiratory chain function was performed using a snap frozen skeletal muscle biopsy sample. The activities of complexes I, II, III and IV were all normal within a frozen homogenate, with no evidence of any major respiratory chain abnormality in this tissue. It was not possible to exclude a defect involving complex V, as this cannot be easily measured in frozen tissue homogenates.

### 3.3 Genetic Findings, *In Silico* Modelling and Functional Studies of ACO2

**FIGURE 3:**
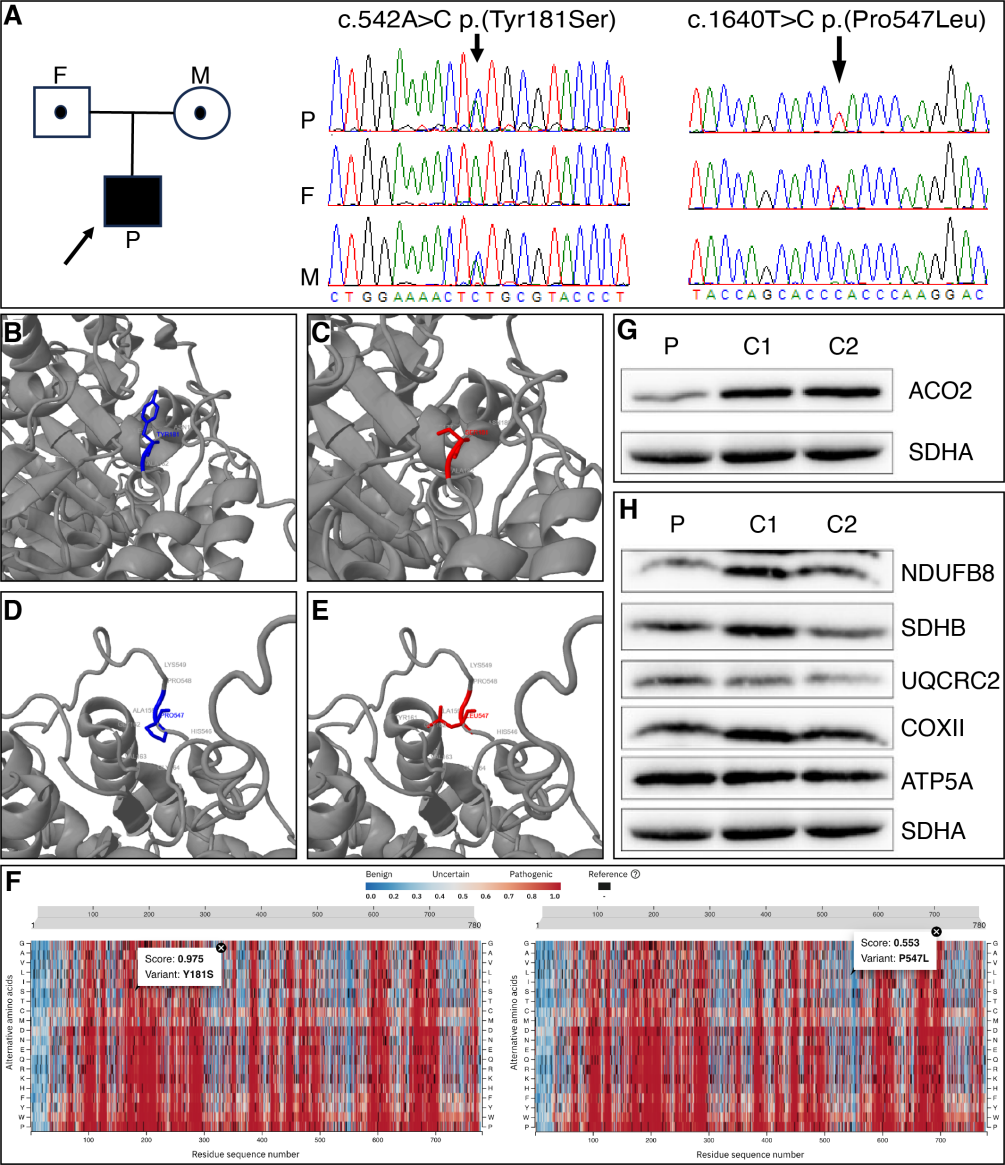
Molecular characterisation and *in silico* modelling of patient *ACO2* variants. (A) *ACO2* family pedigree and sequencing chromatograms with Sanger confirmation of the maternally transmitted c.542A>C p.(Tyr181Ser) *ACO2* variant (left panel) and the paternally transmitted c.1640 T>C p.(Pro547Leu) *ACO2* variant (right panel). Variant nomenclature refers to *ACO2* RefSeq (NM_00198.3). (B–E) *In silico* modelling of *ACO2* variants with Missense3D predicted a deleterious effect of the p.Tyr181Ser substitution, with the wildtype buried tyrosine residue (blue, B) being replaced by an exposed serine residue (red, C). Missense3D did not provide evidence of a deleterious effect due to the p.Pro547Leu substitution, with the proline residue (blue, D) being replaced by a leucine residue (red, **E**). Nearby residues are labelled. (F) AlphaMissense predictions for the patient's *ACO2* variants showed that the p.Tyr181Ser (Y181S, left panel) substitution is scored at 0.975, supportive of pathogenicity, whilst the p.Pro547Leu (P547L, right panel) variant is predicted to have a milder effect, scoring 0.553, falling within the range of uncertain pathogenicity. (G, H) SDS‐PAGE and western blotting demonstrated a marked reduction in ACO2 steady‐state levels in the patient's muscle homogenate lysates compared to two age‐matched controls (G), whilst the patient's steady‐state levels of various different OXPHOS proteins, including NDUFB8 (Complex I), SDHB (Complex II), UQCRC2 (Complex III), COXII (Complex IV) and ATP5A (Complex V), were comparable to those of the controls (H). This aligns with previous results indicating unremarkable respiratory chain enzymology. Images from consecutive immunodetection with ACO2, the OXPHOS cocktail (Abcam), and SDHA, used as a mitochondrial marker, are provided. For clarity, ACO2 is shown separately in (G), while the OXPHOS panel in (H) uses the same SDHA loading control. SDS‐PAGE and immunoblotting were performed in triplicate, with representative data shown. P, patient; F, father; M, mother; C1 and C2, controls.

Variant prioritisation of whole genome sequencing data revealed compound heterozygous *ACO2* variants, a maternally inherited c.542A>C p.(Tyr181Ser) variant and a paternal c.1640 T>C p.(Pro547Leu) variant. Both variants are absent from controls (or at extremely low frequency if recessive) and are not recorded on the population database gnomAD v.4 (Genome Aggregation Database). Given there is currently no available crystal structure for human ACO2, the AlphaFold prediction for the tertiary structure (Q99798) was used to model the functional consequence of the p.Tyr181Ser and p.Pro547Leu residues (Figure 3A–E). Missense3D and AlphaMissense predictions were both supportive of pathogenicity for the p.Tyr181Ser substitution, whilst both tools had less confidence in a deleterious functional consequence associated with the p.Pro547Leu variant, categorising it as of uncertain significance (Figure 3F). SDS‐PAGE and Western blotting of patient muscle biopsy lysates demonstrate a reduction in the steady state levels of ACO2 relative to age and tissue matched controls (Figure 3G). Levels of structural subunits of the OXPHOS system are preserved, consistent with the previous biochemical assessment of OXPHOS complexes (Figure 3H).

Following from our modelling and functional studies, we used the ACMG/AMP (American College of Medical Genetics and Genomics/Association for Molecular Pathology) guidelines [11] and classed both variants as likely pathogenic. The c.542A>C p.(Tyr181Ser) variant was classed as likely pathogenic using the evidence codes PM2 (absent gnomAD v.4), PP4_Moderate (consistent patient phenotype and western blot demonstrating reduced steady state levels of ACO2), PP3 (REVEL & AlphaMissense predictive of a deleterious impact) and PM1_Supporting (variant within aconitase N‐terminal domain close to substrate binding site, within a missense‐constrained domain, predicted to result in conformational amino acid change by 3D protein modelling). The c.1640 T>C p.(Pro547Leu) variant was classed as likely pathogenic using the evidence codes PM2 (absent gnomAD v.4), PP4_Moderate (consistent patient phenotype and Western blot demonstrating reduced steady state levels of ACO2), and PM3 (determined to be *in trans* with c.542A>C in this patient by parental genotyping). *ACO2* homozygous or heterozygous pathogenic variants are associated with infantile cerebellar retinal degeneration (OMIM #614559 [1]) and are described using RefSeq reference sequence NM_001098.3.

### 3.4 *Drosophila* Models of *ACO2* Display Disease Relevant Phenotypes

We developed *Drosophila* fly models to investigate the biological mechanisms that may be involved in the clinical phenotype and pathogenic processes. The *Drosophila* orthologue of *ACO2* is the *mAcon1* gene, which likewise plays a role in the Krebs Cycle within mitochondria. Previous research on aconitase in flies has found effects on locomotor activity, flight ability, lifespan, development, memory and neural degeneration, mirroring some phenotypes observed in humans with defective aconitase [12, 13, 14, 15]. Building upon these earlier investigations, we selectively reduced (*mAcon1* ^*RNAi*^) or increased (*mAcon1* ^*OX*^) the expression levels of *mAcon1* in *Drosophila* and tested these flies in an array of assays.

**FIGURE 4:**
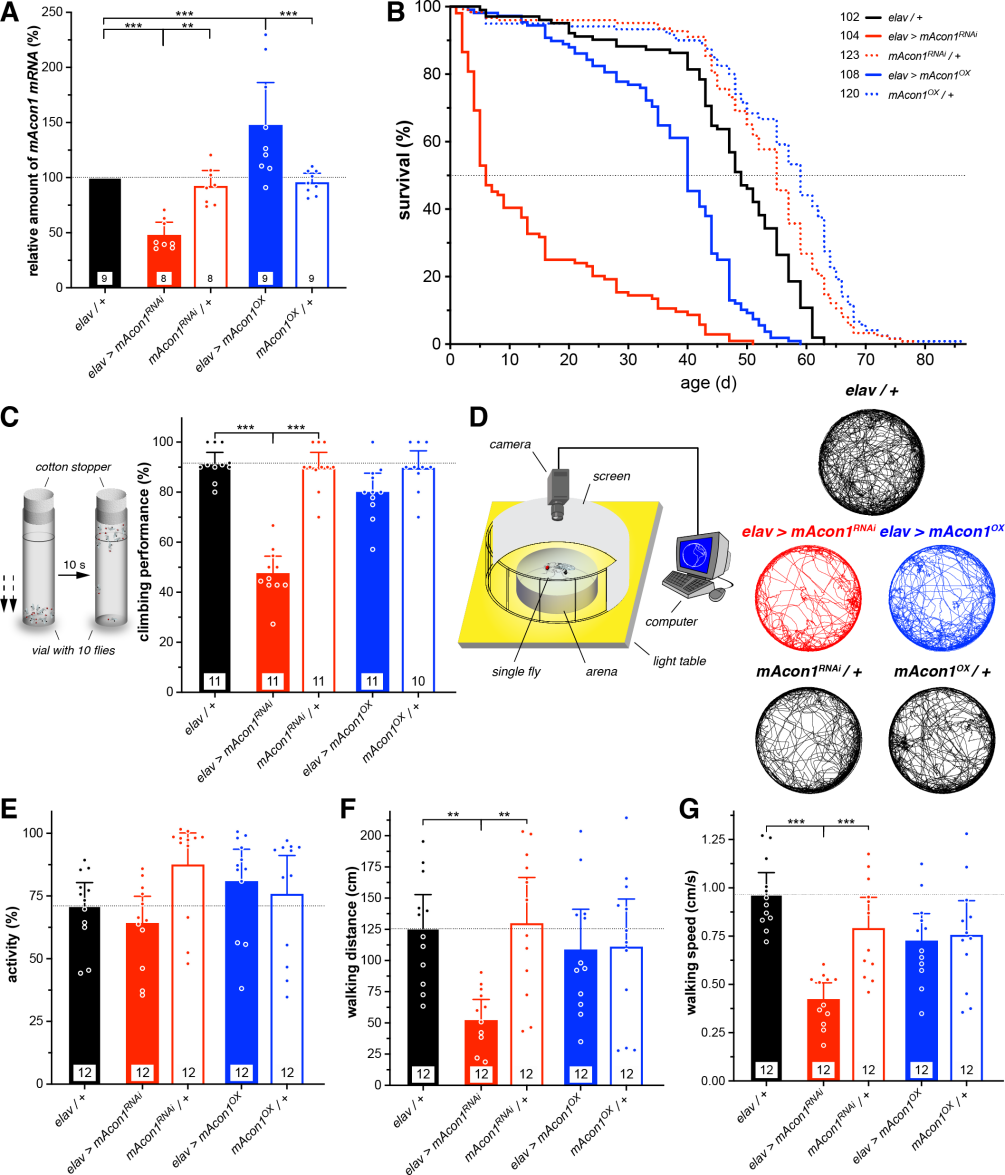
*mAcon1*, the fly homologue of human *ACO2*, shortens lifespan and reduces climbing ability and exploratory behaviour of *Drosophila*. (A) Pan‐neuronal *mAcon1* knock‐down (*elav > mAcon1* ^*RNAi*^, solid red bar) led to a 52% decrease, while *mAcon1* over‐expression (*elav > mAcon1* ^*OX*^, solid blue bar) led to a 48% increase in *mAcon1 mRNA* levels compared to both *Gal4* (black bar) and *UAS* (open bars) controls. n, technical replicates indicated; *N* = three biological replicates with ~40 heads each. (B) *mAcon1* knock‐down (red solid line) dramatically and over‐expression (blue solid line) significantly reduce the lifespan of flies, represented by the proportion of surviving flies over time, compared to controls (*Gal4*, black line; *UAS*, dotted lines). *n*, indicated. (C) In the negative geotaxis assay to assess the climbing proficiency of flies, groups of 10 flies were tapped gently towards the bottom and the number of flies that reached the line within a span of 10 s was counted. Knock‐down of *mAcon1* (solid red bar) resulted in a reduced climbing performance compared to controls. Bars represent means; whiskers represent 95% CI; n represents numbers in bars (groups of 10 flies each). (D) Individual exploratory locomotor behaviour was studied using a solitary fly moving freely within a well‐lit arena (ø 55 mm) for a duration of 3 min. The fly's movements were recorded using a webcam connected to tracking software on a computer. The combined tracks of mutant and control flies (12 flies each) show their exploration patterns, with *mAcon1* knock‐down flies (red traces) covering a smaller area within the arena, tending to avoid the centre. (E–G) The measured parameters identify a decrease in both walking distance (F) and speed (G) for *mAcon1* knock‐down flies (solid red bars) while maintaining a consistent overall activity level (E). Bars, means; whiskers, 95% CI; *n*, numbers in bars.

First, we assessed the efficiency of the transgenes using RT‐qPCR, confirming a 52% reduction in *mRNA* levels for the knockdown (*mAcon1* ^*RNAi*^) and a 48% increase for the overexpression (*mAcon1* ^*OX*^; Figure 4A and Supplementary Table S3). Then, we looked at whether *mAcon1* effects longevity and found that both knockdown and overexpression reduced lifespan (Figure 4B). Interestingly, compared to controls with a lifespan of approximately 50 days, *mAcon1* ^*RNAi*^ flies exhibited significantly accelerated mortality, with a median lifespan of only 6 days, thus affirming the pivotal role of this mitochondrial enzyme. Next, a locomotor assay testing the negative geotaxis behaviour of young flies showed that while *mAcon1* ^*OX*^ showed a mild impairment narrowly failing to reach statistical significance, *mAcon1* ^*RNAi*^ reduced their climbing ability by almost half (Figure 4C). To further investigate locomotor behaviour, we observed spontaneous walking of individual flies within a confined arena. Once more, it was evident that *mAcon1* ^*RNAi*^ flies exhibited impaired locomotion, contrasting with *mAcon1* ^*OX*^ flies. Specifically, individual *mAcon1* ^*RNAi*^ flies walked at a slower pace and covered less distance, despite being active for a comparable duration to the controls (Figure 4D–G, Supplementary Table S3 and Supplementary File S4).

**FIGURE 5:**
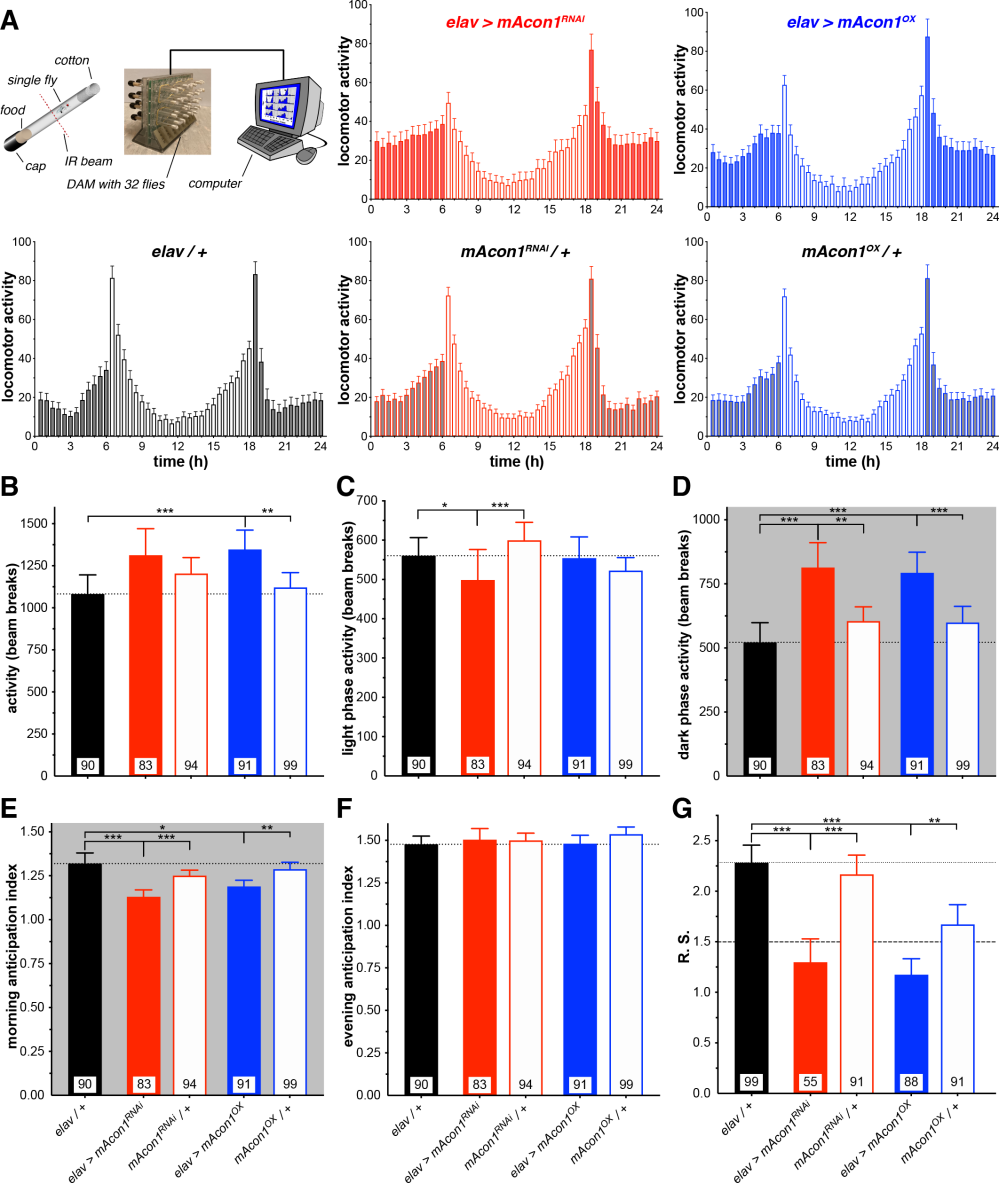
Manipulation of *mAcon1* increases locomotor activity and impairs circadian rhythmicity. (A) Diagram illustrating the setup for automated fly tracking. Each fly is placed individually in a tube containing food and loaded into a DAM monitor placed in an incubator. Inside the monitor, an infrared beam intersects the tube, enabling the recording of fly movements whenever the beam is interrupted. The DAM monitor is connected to a computer that records the total count of beam breaks, providing a measure of the fly's activity. The histograms show the five‐day average daily activity levels of all flies in LD for control and mutant genotypes (filled bars, lights off; open bars, lights on). (B–D) Total activity levels, measured as the average daily number of beam crosses, showed an increase in activity of *mAcon1* over‐expressing flies (B). *mAcon1* knock‐down flies were less active during the day (C) while both manipulations led to increased activity during the night (D). (E–G) Both mutant strains exhibited impaired circadian rhythmicity, leading to a decrease in morning anticipation (E) and weakened circadian rhythm strength (G). However, the evening anticipation (F) remained unaffected. Bars, means; whiskers, 95% CI; *n*, numbers in bars.

**FIGURE 6:**
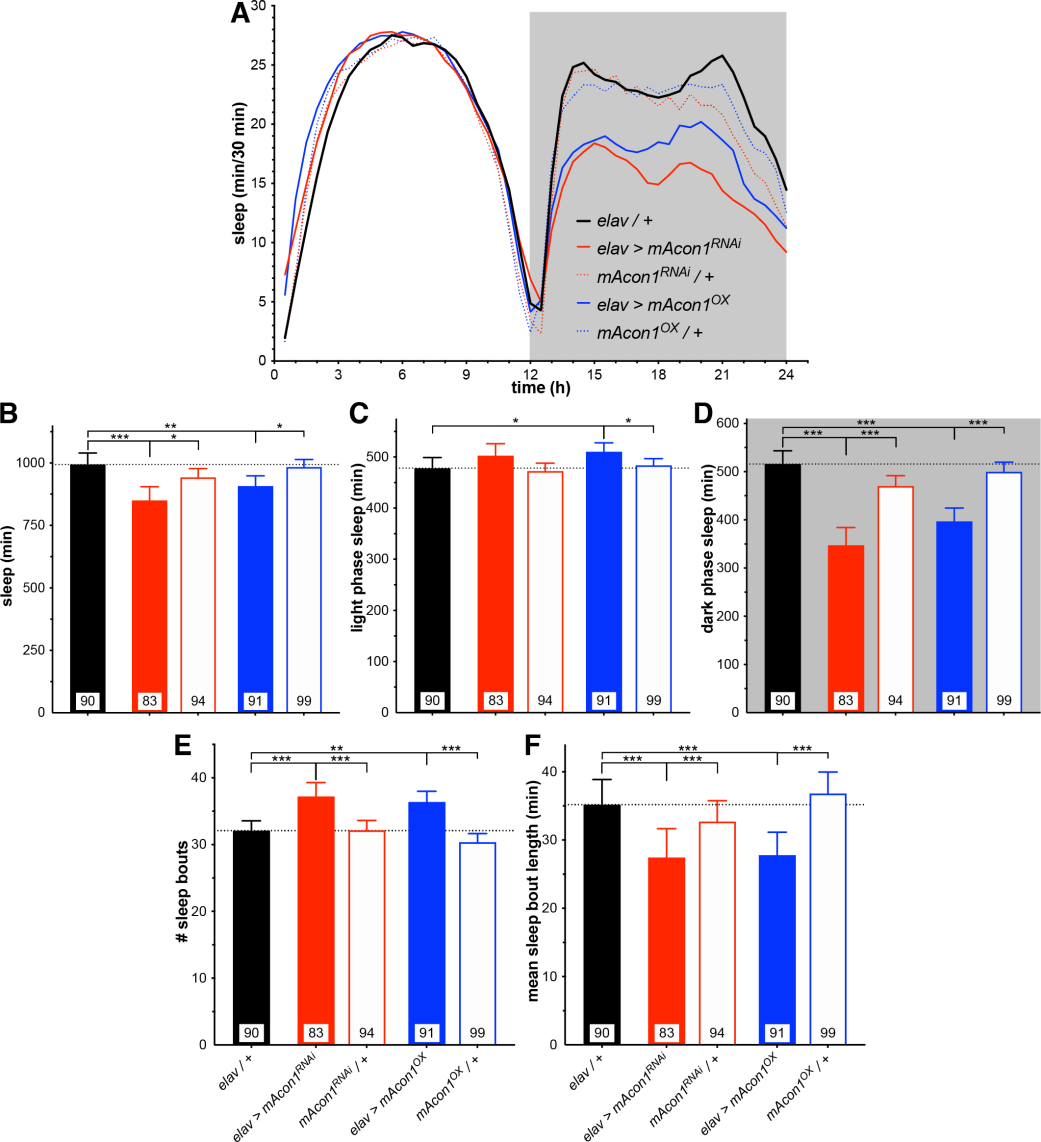
Manipulation of *mAcon1* reduces and fragments sleep. (A) The sleep distribution profiles show the amount of time spent asleep in 30‐min intervals throughout the day, demonstrating a reduction in sleep during the lights off, night period (grey area) for both knock‐down (red trace) and over‐expression (blue trace) of *mAcon1* compared to controls. (B–D) Both mutant strains exhibited decreased total sleep levels (B). Flies over‐expressing *mAcon1*, however, displayed increased daytime sleep (C), while both manipulations resulted in reduced night‐time sleep (D). (E, F) Both mutant strains displayed a modified sleep architecture characterised by an increase in the number of sleep episodes (E) and a decrease in the average length of sleep bouts (F), resulting in more fragmented sleep. Bars, means; whiskers, 95% CI; *n*, numbers in bars.

Subsequently, we investigated flies using the DAM assay to analyse their locomotor patterns and circadian rhythms, which include sleep–wake cycles (Figure 5 and Supplementary Table S3). Under normal 12‐h light and 12‐h dark conditions (LD), the activity of both *mAcon1* ^*RNAi*^ and *mAcon1* ^*OX*^ flies increased, with significance observed only in *mAcon1* ^*OX*^ flies (Figure 5B). Both manipulations showed notably increased activity during the night while *mAcon1* ^*RNAi*^ flies conversely exhibited reduced activity during the day (Figure 5C,D). Furthermore, both genetic manipulations affect the circadian clock, as indicated by decreased morning anticipatory behaviour and rhythm strength under constant dark conditions (DD; Figure 5E,G). Sleep is a vital physiological function for all animals, including flies and humans, and was found to be affected by *mAcon1* manipulations. The same assay revealed that both mutant strains undergo an overall decrease in total sleep duration, particularly at night, while showing a slight compensatory increase in daytime sleep (significant for *mAcon1* ^*OX*^; Figure 6A–D). Notably, both mutants displayed more fragmented sleep patterns characterised by more numerous but shorter sleep episodes (Figure 6E,F and Supplementary Table S3).

**FIGURE 7:**
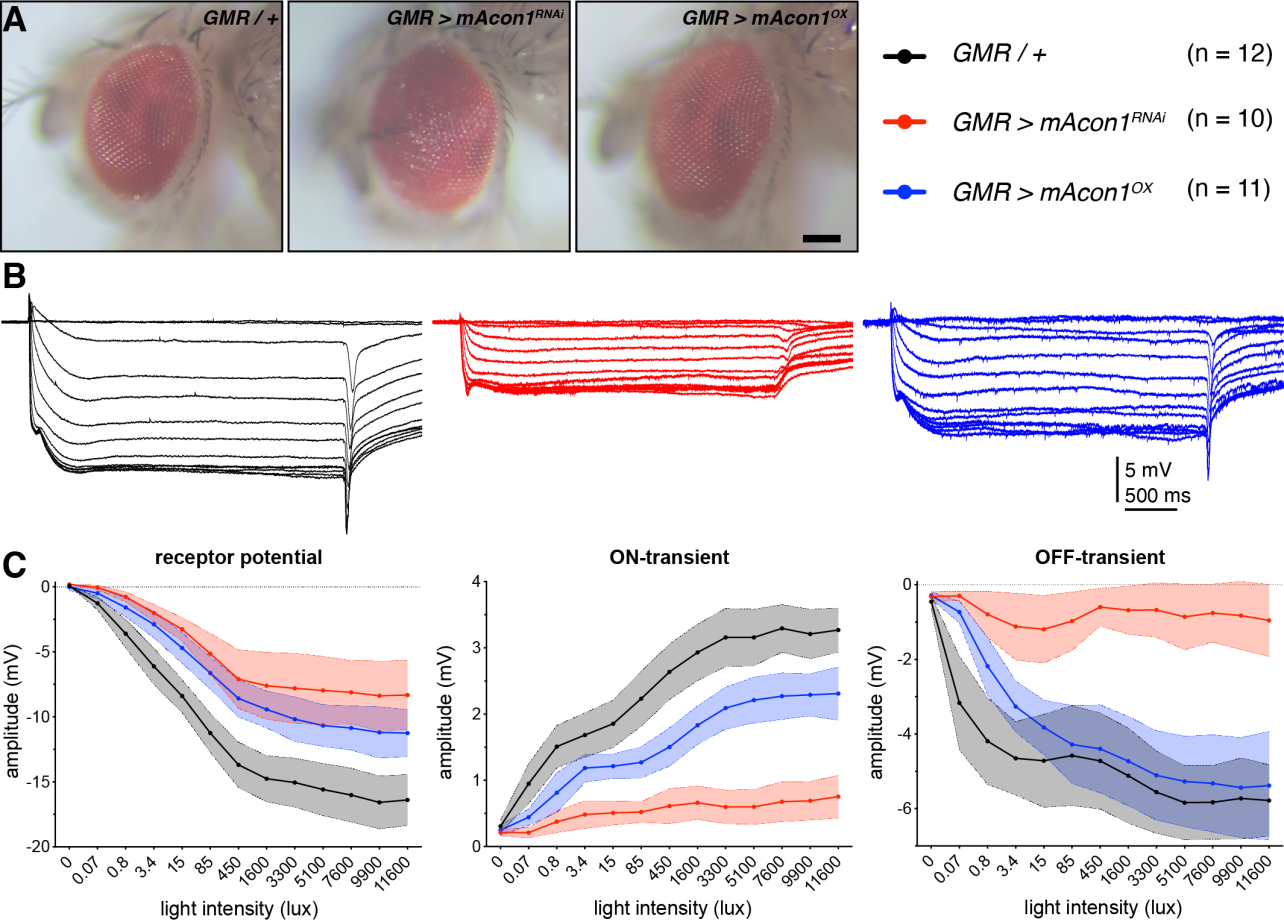
Manipulation of *mAcon1* leads to eye neurodegeneration and reduced electroretinograms. (A) Photographs of the compound eyes of mutant flies were taken to compare them to control flies (*GMR / +*) exhibiting the regular alignment of ommatidia. *mAcon1* knock‐down eyes showed a mild “rough eye” phenotype, which is typically associated with neurodegeneration. Scale bar, 100 μm. (B) Exemplary electroretinogram (ERG) recordings with increasing light intensity stimulation for control and mutant flies. Control flies (black traces) show the typical fly ERG composed of an ON‐transient, sustained photoreceptor response and OFF‐transient. While these responses were reduced for both mutants, *mAcon1* knock‐down flies (red traces) nearly completely lacked both transients. (C) Both knock‐down (red traces) and over‐expression (blue traces) of *mAcon1* resulted in reduced receptor potential (left) compared to controls (black traces) across all stimulus intensities as well as decreased ON‐transients (middle) and OFF‐transients (right). The effects were more pronounced in the knock‐down flies. Lines, means; shaded area, 95% CI; *n*, indicated.

Finally, to investigate the influence of *mAcon1* on eye development and function, we conducted the fly rough eye assay and ERG recordings. Typically, a fruit fly's compound eye surface displays a smooth and regular appearance. However, when *mAcon1* ^*RNAi*^ was expressed in the eyes, we observed deviations from normal eye development, leading to a rough, disorganised appearance and abnormal pigmentation pattern (Figure 7A). We employed the *Drosophila* ERG as an electrophysiological technique to evaluate the functionality of the visual system. The *Drosophila* ERG comprises (1) fast ON transients from laminar neuron hyperpolarisation, triggered by histaminergic photoreceptors (R1‐R6); (2) a sustained corneal‐negative plateau from photoreceptor depolarisation; and (3) OFF transients from laminar neuron repolarisation when histamine release stops. The extracellular field potentials recorded are opposite in polarity to the actual cellular potentials [16]. ERG recordings revealed a reduction in both ERG amplitude and ON/OFF transients, with *mAcon1* ^*RNAi*^ exhibiting a significantly greater impact (Figure 7B,C and Supplementary Table S3). The near complete absence of an ON and OFF response for *mAcon1* ^*RNAi*^ suggests impaired neurotransmission, indicating a defect in synaptic signalling between the photoreceptors and the laminar neurons, while the decreased ERG amplitude points to diminished sensitivity. These experiments collectively demonstrate that manipulating the levels of *mAcon1*, the fly equivalent of human *ACO2*, results in phenotypes relevant to human ICRD.

## 4 Discussion

This study combines a clinical case study of a paediatric patient with *ACO2* variants and experimental research using a *Drosophila* animal model to explore the underlying mechanisms of infantile cerebellar‐retinal degeneration (ICRD). By integrating clinical observations with targeted manipulations of the fly ortholog *mAcon1*, we provide a comprehensive approach to understanding *ACO2*‐related pathology.

The patient's presentation adds to the spectrum of *ACO2*‐related disorders, marked by an initially subtle clinical course that evolved into distinct neurological and ophthalmological deficits over time. The patient's symptoms and neuroimaging findings are consistent with ICRD, characterised by hypotonia, developmental delay, optic atrophy and progressive MRI changes, including cerebellar atrophy and dentate nuclei hyperintensities (Figure 1). Extensive diagnostic evaluations, including MRI and visual electrophysiology, were consistent with previously reported findings and underscore the critical role of MRI in monitoring disease progression. Genetic analysis revealed compound heterozygosity for two *ACO2* variants (Tyr181Ser and Pro547Leu), both initially classified as variants of uncertain significance. While their pathogenicity was unclear, their absence from population databases and the consistency of the patient's phenotype with known *ACO2*‐related disorders suggested a causative link. *In silico* modelling predicted a deleterious effect, particularly for the p.Tyr181Ser residue, and indeed SDS‐PAGE and Western blotting of patient skeletal muscle biopsy lysates demonstrated reduced steady state levels of ACO2, consistent with instability of the ACO2 protein, thereby providing unequivocal support for pathogenicity (Figure 3). Based on this, we reclassified both variants as likely pathogenic under ACMG guidelines [11]. Notably, heterozygous pathogenic ACO2 variants have previously been associated with isolated optic atrophy [4]. Together, these findings align with the few documented cases of ICRD and support a possible connection between *ACO2* variants and neurodegeneration, consistent with a recent review [17], which associates aconitase dysfunction with neurodegeneration. Our study further supports this link by showing that ACO2 dysfunction leads to cerebellar atrophy and ataxia. ACO2 is a mitochondrial enzyme essential for the citric acid cycle, playing a critical role in the isomerisation of citrate to isocitrate and maintaining normal metabolic function through its iron–sulphur cluster. Mutations in the *ACO2* gene disrupt this enzymatic activity, leading to altered metabolite levels, specifically increased glutamate and reduced alpha‐ketoglutarate, which are believed to contribute to the neurological symptoms observed in affected patients [18].

Amongst the previously described cases of ICRD worldwide, most presented with symptoms of neurodegeneration including hypotonia, seizures and optic atrophy usually before the age of 1. Unlike other reported cases, this case developed with subtle features in infancy, becoming more apparent clinically and radiologically over time. This patient underwent holistic investigation to assess for cerebellar and retinal degradation. While laboratory investigations displayed no metabolic abnormalities and only mild non‐specific myopathic features (Figure 2), changes were seen in the MRI scans of the brain and visual electrophysiology. This was consistent with previously reported cases where brain MRI changes, while not always seen when symptoms presented, were noticed in late childhood or teenage years. Interestingly, ACO2 deficiency has also been linked to an increased risk of movement disorders, such as Parkinson's disease [19]; however, this association is based on adult data, which may not be directly applicable to paediatric cases. These clinical and radiological assessments were regularly followed up with clinical reviews.

To further investigate the impact of *ACO2* variants on neurological phenotypes, we used *Drosophila melanogaster* as a model organism, manipulating the fly ortholog *mAcon1* through RNA interference (*RNAi*) knock‐down and over‐expression. These experiments aimed to explore the effects of altered gene function, given the uncertain impact of the *ACO2* variants on enzyme activity. Previously, using a similar method, we demonstrated that pan‐neuronal over‐expression or knock‐down of the *Drosophila* ortholog of *ADD3*, known as *hts*, could replicate certain aspects of the human disease [20]. Given that the human phenotype exhibits cerebellar and musculoskeletal locomotor deficits, we examined neuronal misexpression of fly *mAcon1* and identified disease‐relevant phenotypes. Both knock‐down and over‐expression of *mAcon1* affected lifespan and locomotor behaviour (Figure 4), indicating the necessity of a balanced gene product level for normal mitochondrial function. Consistent with previous studies [12], knock‐down of fly *mAcon1* resulted in more severe phenotypes in our assays. Sleep and circadian rhythms in flies are also affected (Figures 5 and 6), suggesting broader neurological impacts of *ACO2* variants that warrant further investigation in human patients. It remains unclear whether lifespan is affected in humans as well. Furthermore, the human patient exhibited ERG deficits, which were mirrored in flies; both increased and decreased *mAcon1* levels resulted in visual processing defects, with *RNAi* knock‐down exacerbating the impairment (Figure 7). Intriguingly, neurodegeneration was observed in fly eyes, potentially resembling the loss of cerebellar volume and optic nerve atrophy observed in the patient. In line with fly knockouts being lethal [12], some effects of *mAcon1* knock‐down were severe, including reduced lifespan, climbing ability and ERG responses, while over‐expression generally led to milder impairments.

In summary, we have successfully established an animal model for *ACO2*, demonstrating that fly orthologs of human genes linked to cerebellar‐retinal degeneration can replicate relevant disease phenotypes. The striking parallels between human and fly models underscore the value of *Drosophila* in studying *ACO2*‐related neurodegeneration and provide insights into potential cellular mechanisms, such as disrupted mitochondrial function and neurotransmission defects. While our study highlights the phenotypic consequences of *ACO2* variants, limitations include the rarity of these variants, restricting comparative patient data, and physiological differences between flies and humans, which warrant caution in extrapolating findings. Additionally, further validation in mammalian systems is necessary to definitively establish pathogenicity, and preclinical studies are required to assess the efficacy and safety of potential therapeutic interventions. Excitingly, recent advances now enable the use of patient‐derived pluripotent stem cells as a complement to animal models, opening new research avenues while offering further pathological insights and facilitating therapeutic validation [21, 22]. Future research should focus on targeted therapeutic interventions, such as genetic rescue experiments or drug screening in the fly model and stem cell‐based studies, to identify potential treatments for *ACO2* deficiency. Our animal experiments provide a foundation for understanding the neurological phenotype, elucidating the biological processes associated with *ACO2*‐related disorders and exploring strategies for managing or preventing these conditions.

## 5 Conclusion

This study uniquely combines clinical analysis with *Drosophila* modelling to explore the biological underpinnings of *ACO2* variants, expanding our understanding of *ACO2*‐related disorders. By linking specific genetic variants to phenotypic outcomes in both a paediatric patient and a fly model, we provide valuable insights into the effects of these variants and highlight the need for early genetic screening in patients with ICRD‐like symptoms (e.g., hypotonia, motor delays, visual impairment). We propose Sanger sequencing to identify compound heterozygous *ACO2* variants, supported by the gene's eight ‘green status’ whole genome sequence panels in the UK PanelApp: Optic Neuropathy; Retinal Disorders; Inborn Errors of Metabolism; Undiagnosed Metabolic Disorders; Mitochondrial Disorders; Possible Mitochondrial Disorder—Nuclear Genes; Intellectual Disability and Severe Paediatric Disorders [6]. The *Drosophila* model of *mAcon1* serves as a vital tool for further investigating the neurological consequences of *ACO2* variants and developing potential interventions, and may in future be used as a way to functional screen for damaging variants and help discovery of rare neurodevelopmental diseases [9]. This combined approach highlights key aspects of mitochondrial dysfunction and its impact on neurodevelopment, enhancing our understanding of *ACO2*‐related disorders and offering a promising platform for future research into targeted therapies and preventive strategies for ACO2 deficiency.

## Author Contributions

**Edgar Buhl:** conceptualisation, methodology, formal analysis, investigation, writing – original draft, writing – review and editing, visualisation, project administration, funding acquisition. **Suchika Garg:** investigation, writing – original draft, writing – review and editing, visualisation. **Marie Monaghan:** conceptualisation, investigation, methodology, writing – original draft, writing – review and editing. **Amy Preston:** investigation. **Marcus Likeman:** investigation, visualisation. **Julianne Dare:** investigation. **Julie Evans:** investigation. **Lucie S. Taylor:** investigation. **Ian Berry:** investigation. **Kathryn Urankar:** investigation, visualisation. **Paul G. D. Spry:** investigation. **Cathy Williams:** investigation. **Robert W. Taylor:** supervision. **Charlotte L. Alston:** investigation, writing – original draft, visualisation. **James J. L. Hodge:** conceptualisation, methodology, writing – review and editing, supervision, project administration, funding acquisition. **Anirban Majumdar:** conceptualisation, methodology, investigation, writing – original draft, writing – review and editing, visualisation, supervision, project administration.

## Conflicts of Interest

The authors declare no conflicts of interest.

## Peer Review

The peer review history for this article is available at https://www.webofscience.com/api/gateway/wos/peer‐review/10.1111/cge.14745.

## Supporting information


**Data S1.** Supporting Information.

**Table S2.** Patient Electroretinogram (ERG) and Visual Evoked Potentials (VEP).

**Table S3.** Data table for *Drosophila* experiments.

**Data S2** Supporting Information.

## Data Availability

The data that supports the findings of this study are available in the supporting Information of this article.
