# Supplementary material for: Infantile Cerebellar‐Retinal Degeneration Associated With Novel ACO2 Variants: Clinical Features and Insights From a Drosophila Model
Source: Clin Genet. 2025 Apr 10;108(3):266–78. doi: 10.1111/cge.14745 (PMC12319146; doi:10.1111/cge.14745)
Supplement: Supplementary file 1 — Data S1. Supporting Information. [file CGE-108-266-s003.pdf]

Supplementary Methods S1:

1 Clinical details and Informed Consent

The clinical details were collected systematically through paediatric neurology clinic reviews on a yearly basis over a period of six years. Basic historical and demographic details were collected, and a full clinical examination was conducted and recorded. Parents of the patient have given full, written informed consent for the writing and dissemination of this case including clinical history, investigation results and imaging. Institutional and ethical approval was sought, and it was deemed not necessary for the purposes of publication.

2 Investigations

In view of the clinical concerns around hypotonia and developmental delay, the child was investigated in a standard manner. Initially MRI scans were performed from the age of one to explore central causes of the hypotonia. With the development of subsequent visual impairment further MRI scans were performed. In addition, a range of tests, including metabolic screens, were carried out on peripheral blood and cerebrospinal fluid. At three years of age, a muscle biopsy together with further histochemical and immunohistochemistry analysis was carried out to further investigate the emerging clinical features. Biochemical assessment of mitochondrial respiratory chain function was performed using a snap frozen skeletal muscle biopsy sample using previously reported methodology [1]. At six years old, he developed visual impairment, which was investigated using visual electrophysiology, including visual evoked potentials (VEP) recorded from a single midline occipital scalp electrode and electroretinography (ERG) performed with a xenon arc discharge tube stimulator and lower eyelid surface-mounted electrodes.

3 Molecular Genetic Investigations

The patient and his parents were recruited for trio whole genome sequencing as part of the 100,000 Genomes Project [2]. Prioritised variants were confirmed by Sanger sequencing and classified according to ACGS [3] and ACMG [4] guidelines. *In silico* modelling was performed using AlphaFold and Missense3D [5], using the AlphaFold prediction for human aconitase (Q99798).

4 Assessment of ACO2 and OXPHOS protein stability in patient-derived fibroblasts

Investigation of steady-state levels of ACO2 and mitochondrial OXPHOS complex subunits was performed using SDS–polyacrylamide gel electrophoresis (SDS–PAGE) using patient and age-matched control muscle homogenates, as previously described [6]. Western blot analysis was carried out using primary antibodies conjugated against

various OXPHOS subunits, each used a 1 in 1,000 dilution (ACO2 (Abcam ab129069), the total OXPHOS Human WB Antibody Cocktail (ab110411, comprising NDUFB8, UQCRC2, COX1, COXII and ATP5A)). SDHA (Abcam ab14715) was used as a loading control. HRP-conjugated secondary antibodies (Dako anti-rabbit (P0399) used at 1 in 3,000 dilution and anti-mouse (P0260) used at 1 in 2,000 dilution) facilitated imaging using enhanced chemiluminescence on a BioRad ImageQuant GelDoc platform.

5 *Drosophila* animal model of *ACO2*

5.1 Fly husbandry

Flies were raised with a 12h:12h light:dark (LD) cycle with lights on at ZT 0 (Zeitgeber Time) in standard vials (95 mm, ø 25 mm) on standard *Drosophila* medium (0.75% agar, 5.5% glucose, 5.0% yeast, 3.5% wheat flour, 0.4% propionic acid, 2.5% nipagin 10%, 1% penicillin/streptomycin) at 25°C and collected between 2-5 days post eclosion. The following strains were used in this study and obtained from the Bloomington *Drosophila* Stock Center (Indiana University, IN, USA) or as indicated: *UAS-mAcon1-RNAi* (BL24751), *UAS-mAcon1* (BL24752), *elav-Gal4* (BL8765), *GMR-Gal4* (BL9146), *Canton-S w-* (gift from Dr Scott Waddell, University of Oxford, UK). The *mAcon1* knock-down (RNAi) or over-expressor transgenes were expressed under the control of *GMR-Gal4* for eye imaging and recording and *elav-Gal4* (BL8765) for all other assays; controls were generated by crossing driver and responder lines to *Canton-S w-*. All chemicals were purchased from Sigma-Aldrich (Gillingham, UK).

5.2 RT-qPCR

Relative measure of *mAcon1* expression levels was assessed by RT-qPCR. Two to five days old flies were anesthetised with CO_2 and decapitated, obtaining three biological replicates with ~40 heads each. Total *RNA* was extracted from head lysates by organic phenol/chloroform method using TRIzol reagent (Invitrogen). *RNA* was stored at -80 °C or used immediately for downstream reactions. *RNA* quantification was carried out in Nanodrop spectrophotometer (Thermo Scientific) and reverse transcription was carried out using RevertAid First Strand cDNA Synthesis Kit (Thermo Scientific) following manufacturer's instructions, with 1500 ng of *RNA* as template and Oligo(dT) as primer to amplify total *mRNA*. *cDNA* samples were stored at -80 °C or used immediately for qPCR reactions.

Quantitative PCR reactions were carried out in QuantStudio 3 Real-Time PCR system (Applied Biosystems) using PowerUp™ SYBR™ Green Master Mix (Applied Biosystems). The primers used to amplify *mAcon1 mRNA* were as follows: *mAcon1*-Fwd 5'-GGACCATTCACTCCCGATTT -3' and *mAcon1*-Rev 5'-GTTGGTGCAAGAGCCAATAAG-3' with a PCR product expected size of 111 bp. As a housekeeping gene,

the following primers for α-*tubulin mRNA* were used: *α-tub*-Fwd 5’-CCTCGAAATCGTAGCTCTACAC-3’ and *α-tub*-Rev 5’-CAGCCTGACCAACATGGATA-3’. The expected PCR product size was 112 bp. To activate UDG, two minutes at 50°C was used, then two minutes at 95°C was used to activate DNA polymerase, followed by 40 cycles of 15 s at 95°C, and one minute at 60°C for the anneal/extension step. At the end of the experiment, a temperature ramp from 60°C to 95°C was performed for melting curve analysis and the curve's shape was examined for overlaps or double peaks to ensure the correct amplicons were present. Quantification for each genotype and each gene was carried out using the $2^{(-\Delta\Delta Ct)}$ method and data expressed as a percentage of change.

5.3 Longevity assay

Ten mated females were transferred to a vial containing standard food and maintained at 25°C and 70% humidity throughout. Only mated females were used since the activity of the larvae prevents the food from drying out too quickly and females live significantly longer than males. For each genotype at least ten concurrent replicates were used. Flies were transferred to fresh food and deaths scored every 2-3 days.

5.4 Negative geotaxis climbing assay

Male flies were collected and acclimatised in the test vial (95 mm, ø 25 mm) for 30 min at room temperature (21°C) and experiments performed during the day (ZT2-3). Only male flies were used to avoid any potential gender effect on this assay. Using the negative geotaxis reflex of *Drosophila*, groups of ten flies were gently tapped to the bottom and the number of flies counted that climbed to above seven cm within ten seconds. For each genotype at least ten replicates were used and climbing performance was calculated as the average percentage of flies meeting these criteria.

5.5 Video tracking of fly locomotor activity

To record and analyse individual fly locomotor behaviour, single flies of either sex were placed in a flat 55 mm round arena on a light table and recorded from above with a standard webcam (Tecknet Full HD 1080p; Tecknet Ltd, Liverpool, UK) and Buridan tracker software [7]. Fly trajectories were recorded at ZT1-4 and room temperature (22°C) for three min. Data were analysed using an Excel Visual Basic program (Fly Analyses; [8]) and percentage of movement activity, mean walking distance and mean walking velocity calculated.

5.6 Sleep, activity and circadian behaviour

Analysis of sleep, locomotor activity and circadian rhythm strength was performed using the *Drosophila* Activity Monitor system (DAM2, Trikinetics Inc., Waltham, MA, USA) and analysed in MATLAB using the Sleep and Circadian Analysis MATLAB Program (SCAMP; [9]. Individual entrained male flies were placed in DAM tubes with a small amount of standard food. Male flies were used, as their behaviour is more stable in this assay while female activity varies depending on egg laying and larval activity. The DAM monitors were located inside a light- and temperature-controlled incubator (Percival Scientific Inc., Perry, IA, USA) where the fly's activity was monitored for five days in 12h:12h light:dark cycles (LD) followed by five days under constant darkness (DD) at 25°C and 70% relative humidity. Data were collected from three or four independent experiments for each genotype and pooled. Flies that had died before the end of the experiment were removed from the analysis.

Locomotor activity was collected in one-minute bins and sleep was defined as five or more minutes of inactivity [10,11]. Activity and sleep measurements were averaged over the five days of LD, and further split into the day (i.e., when lights were on) and night (i.e., lights off) components. Parameters analysed were activity as number of beam crosses, time asleep, number of sleep episodes and mean sleep episode duration. Anticipation indices were calculated from the activity of flies across the five days of LD by taking the ratio of the average activity three hours before the light transition over the six hours before transition. The morning anticipation index (MAI) is thus the ratio of ZT22-24 over ZT19-24 and evening anticipation (EAI) the ratio of ZT10-12 and ZT7-12. Circadian rhythmic statistics (R.S.), a measure of the strength of the circadian behaviour, was calculated by autocorrelation analysis of the activity record for the five days in DD. Flies with a RS > 1.5 were defined as rhythmic. Because RS cannot be negative, flies with a calculated RS < 0 were assigned a power of 0 for subsequent analysis.

5.7 Eye imaging and ERG recordings

To test for neurodegeneration in the eye, flies of either sex were anesthetised by CO_2 and images of the eyes were taken with a Zeiss AxioCam 208 colour camera attached to a stereomicroscope (Zeiss SteREO Discovery.V8, 8× magnification).

For the electroretinogram recordings a single fly was immobilised ventral side down on a Sylgard dish using insect pins, with an additional pin used to fix its head in place. A white LED used for stimulation was placed approximately six mm away from the fly. A fine tungsten pin served as the recording electrode and was inserted into the fly's eye, directed towards the LED. The reference electrode was placed in the abdomen of the fly. The entire setup was covered, and the fly was allowed to dark adapt for 15 min. The experiment was conducted under red light illumination, data acquired using an AxoClamp 2B amplifier (30 kHz filter) and PowerLab 2/20 digitiser. The LED

was controlled through an ISO-STIM 01D, with each light pulse lasting three seconds in 13 steps of 0.01 V, ranging from 0.21 V to 0.33 V (equivalent to 0-11600 lux as measured at the fly's eye). To minimise noise in the traces, the recordings were averaged over two stimulations each and the signal was allowed to return to baseline between each pulse. The baseline was calculated as the average of the pre-pulse recording and the receptor potential by subtracting the baseline from the average recorded at the end of the pulse before the lights were turned off. The ON transient response was determined as the maximum response after lights were turned on, minus the baseline. Likewise, the OFF transient response was calculated as the minimum response after lights were turned off, minus the average response before lights were turned off.

5.8 Data analysis and statistics

All statistical analyses were performed using GraphPad Prism 9 (GraphPad Software Inc.) and figures were arranged in Adobe Illustrator (Adobe Systems Inc.). All data were scrutinised to check they met the assumptions of parametric analyses using the D'Agostino-Pearson test with alpha = 0.05. For the longevity assay a Logrank (Mantel-Cox) comparison of survival curves, for ERG recordings a two-way ANOVA with Dunnett's *post hoc* tests and for all other comparisons, either one-way ANOVA with Šídák's *post hoc* tests or Kruskal-Wallis with Dunn's *post hoc* tests compared to *Gal4* and to respective *UAS* controls were performed. Details of statistical tests used are in Table S1. Statistical levels are denoted as following *p<0.05, **p<0.01 and ***p<0.001. Longevity data are presented as medians and all other data as means and 95% confidence intervals (95% CI).

References

1. Frazier AE, Vincent AE, Turnbull DM, Thorburn DR, Taylor RW. Assessment of mitochondrial respiratory chain enzymes in cells and tissues. In: Pon LA, Schon EA, eds. *Methods in Cell Biology.* Vol 155. Academic Press; 2020:121-156.
2. Caulfield M, Davies, J., Dennys, M., Elbahy, L., Fowler, T., Hill, S., Hubbard, T., Jostins, L., Maltby, N., Mahon-Pearson, J., McVean, G., Nevin-Ridley, K., Parker, M., Parry, V., Rendon, A., Riley, L., Turnbull, C., Woods, K. The National Genomic Research Library. *Genomics England.* 2017;v5.1.
3. Durkie M, Cassidy E-J, Berry I, et al. ACGS Best Practice Guidelines for Variant Classification in Rare Disease 2024. 2024; https://www.acgs.uk.com/media/12533/uk-practice-guidelines-for-variant-classification-v12-2024.pdf. Accessed 01/11/2024.
4. Richards S, Aziz N, Bale S, et al. Standards and guidelines for the interpretation of sequence variants: a joint consensus recommendation of the American College of Medical Genetics and Genomics and the Association for Molecular Pathology. *Genet Med.* 2015;17(5):405-424.
5. Ittisoponpisan S, Islam SA, Khanna T, Alhuzimi E, David A, Sternberg MJ. Can predicted protein 3D structures provide reliable insights into whether missense variants are disease associated? *J Mol Biol.* 2019;431(11):2197-2212.
6. Oláhová M, Hardy SA, Hall J, et al. LRPPRC mutations cause early-onset multisystem mitochondrial disease outside of the French-Canadian population. *Brain.* 2015;138(Pt 12):3503-3519.
7. Colomb J, Reiter L, Blaszkiewicz J, Wessnitzer J, Brembs B. Open source tracking and analysis of adult *Drosophila* locomotion in buridan's paradigm with and without visual targets. *PLOS One.* 2012;7(8):42247.
8. Stone B, Burke B, Pathakamuri J, Coleman J, Kuebler D. A low-cost method for analyzing seizure-like activity and movement in *Drosophila*. *JoVE.* 2014;19(84):51460.
9. Donelson NC, Kim EZ, Slawson JB, Vecsey CG, Huber R, Griffith LC. High-resolution positional tracking for long-term analysis of *Drosophila* sleep and locomotion using the "tracker" program. *PlOS One.* 2012;7(5):e37250-e37250.
10. Shaw PJ, Cirelli C, Greenspan RJ, Tononi G. Correlates of sleep and waking in *Drosophila melanogaster*. *Science.* 2000;287(5459):1834-1837.
11. Hendricks JC, Finn SM, Panckeri KA, et al. Rest in *Drosophila* Is a Sleep-like State. *Neuron.* 2000;25(1):129-138.
