# Supplementary material for: Infantile Cerebellar‐Retinal Degeneration Associated With Novel ACO2 Variants: Clinical Features and Insights From a Drosophila Model
Source: Clin Genet. 2025 Apr 10;108(3):266–78. doi: 10.1111/cge.14745 (PMC12319146; doi:10.1111/cge.14745)
Supplement: Supplementary file 2 — Table S2. Patient Electroretinogram (ERG) and Visual Evoked Potentials (VEP). [file CGE-108-266-s001.pdf]

Supplementary Table S2: Patient Electroretinogram (ERG) and Visual Evoked Potentials (VEP).

Light Adapted Retinal Responses

		Normative values		Patient data	
	**unit**	**median**	**range**	**Right Eye**	**Left Eye**
Cone					
a wave latency	msec	10	8 to 13	**12**	**10**
a wave amplitude	µV	-8.5	-4.5 to -32.3	**-6.9**	**-5.2**
b wave latency	msec	31	28 to 36	**29**	**30**
b wave amplitude	µV	19.3	13.5 to 54.9	**17.4**	**14.1**

Cortical Responses to Flash
NMR = no measurable response

		Normative values		Patient data	
	**unit**	**Mean**	**range**	**Right Eye**	**Left Eye**
Light Adapted Flash					
P2 latency	msec	92	63 to 191	**NMR**	**142**
P2 amplitude	µV	17.5	9.8 to 25.4		**2.6**

COPY

(1) Normal ranges constitute range between 2.5th and 97.5th percentile. For EOG and cortical responses, a normal distribution is assumed, with empiric data used for all retinal responses.
(2) Ranges stated are preliminary and from limited sample size.
