# Supplementary material for: Infantile Cerebellar‐Retinal Degeneration Associated With Novel ACO2 Variants: Clinical Features and Insights From a Drosophila Model
Source: Clin Genet. 2025 Apr 10;108(3):266–78. doi: 10.1111/cge.14745 (PMC12319146; doi:10.1111/cge.14745)
Supplement: Supplementary file 3 — Table S3. Data table for Drosophila experiments. [file CGE-108-266-s004.pdf]

Supplementary Table S3: Data table for *Drosophila* experiments.

genotype		*elav / +*	*elav >* $mAcon1^{RNAi}$	$mAcon1^{RNAi}$ */ +*	*elav >* $mAcon1^{OX}$	$mAcon1^{OX}$ */ +*
**relative *mAcon1 mRNA* (%)**		100	48.1 (13.7)	92.5 (16.7)	147.9 (49.9)	95.9 (10.5)
n		*9*	*8*	*8*	*9*	*9*
significance (p)	vs. *Gal4*		< 0.001		< 0.001	
(1way ANOVA)	vs. *UAS*		0.001		< 0.001	
**median lifespan (d)**		49	6	55	40	59
n		*102*	*104*	*123*	*108*	*120*
significance (p)	vs. *Gal4*		< 0.001		< 0.001	
(M-C test)	vs. *UAS*		< 0.001		< 0.001	
**climbing performance (%)**		91.5 (4.4)	47.7 (6.8)	89.9 (6.0)	80.1 (7.5)	89.8 (6.8)
n		*11*	*11*	*11*	*11*	*10*
significance (p)	vs. *Gal4*		< 0.001		0.02	
(1way ANOVA)	vs. *UAS*		< 0.001		0.09	
**walking**						
n		*12*	*12*	*12*	*12*	*12*
**active period (%)**		71.0 (9.4)	64.2 (10.7)	87.6 (12.5)	80.9 (12.7)	75.9 (15.3)
significance (p)	vs. *Gal4*		0.87		0.62	
(1way ANOVA)	vs. *UAS*		0.02		0.95	
**distance (cm)**		125.7 (27.1)	52.3 (16.5)	129.9 (36.6)	108.8 (32.3)	111.1 (38.2)
significance (p)	vs. *Gal4*		0.002		0.87	
(1way ANOVA)	vs. *UAS*		0.001		> 0.99	
**speed (cm/s)**		0.96 (0.11)	0.42 (0.08)	0.79 (0.16)	0.73 (0.10)	0.76 (0.18)
significance (p)	vs. *Gal4*		< 0.001		0.04	
(1way ANOVA)	vs. *UAS*		< 0.001		> 0.99	
**sleep**						
n		*90*	*83*	*94*	*91*	*99*
**total sleep (min)**		994 (46)	850 (55)	943 (35)	908 (41)	984 (30)
significance (p)	vs. *Gal4*		< 0.001		0.006	
(K-W test)	vs. *UAS*		0.02		0.04	
**day sleep (min)**		478 (21)	503 (23)	472 (16)	510 (17)	484 (13)
significance (p)	vs. *Gal4*		> 0.99		0.03	
(K-W test)	vs. *UAS*		0.07		0.01	
**night sleep (min)**		516 (27)	347 (37)	470 (22)	397 (28)	500 (20)
significance (p)	vs. *Gal4*		< 0.001		< 0.001	
(K-W test)	vs. *UAS*		< 0.001		< 0.001	

**number of sleep bouts**		32.1 (1.5)	37.2 (2.1)	32.1 (1.5)	36.4 (1.6)	30.4 (1.3)
significance (p) *(K-W test)*	vs. *Gal4*		< 0.001		0.003	
	vs. *UAS*		< 0.001		< 0.001	
**mean sleep bout (min)**		35.2 (3.7)	27.4 (4.2)	32.7 (3.1)	27.8 (3.4)	36.8 (3.1)
significance (p) *(K-W test)*	vs. *Gal4*		< 0.001		< 0.001	
	vs. *UAS*		< 0.001		< 0.001	
**activity**						
n		*90*	*83*	*94*	*91*	*99*
**total activity**		1082 (114)	1313 (157)	1204 (95)	1347 (115)	1122 (87)
significance (p) *(K-W test)*	vs. *Gal4*		0.02		< 0.001	
	vs. *UAS*		> 0.99		0.008	
**day activity**		560 (46)	499 (78)	600 (46)	554 (54)	523 (33)
significance (p) *(K-W test)*	vs. *Gal4*		0.01		> 0.99	
	vs. *UAS*		< 0.001		> 0.99	
**night activity**		522 (77)	814 (97)	605 (56)	793 (80)	599 (63)
significance (p) *(K-W test)*	vs. *Gal4*		< 0.001		< 0.001	
	vs. *UAS*		0.007		< 0.001	
**morning anticipation**		1.32 (0.06)	1.13 (0.04)	1.25 (0.03)	1.19 (0.04)	1.29 (0.04)
significance (p) *(K-W test)*	vs. *Gal4*		< 0.001		0.02	
	vs. *UAS*		< 0.001		0.007	
**evening anticipation**		1.48 (0.05)	1.50 (0.07)	1.50 (0.04)	1.48 (0.05)	1.54 (0.04)
significance (p) *(K-W test)*	vs. *Gal4*		> 0.99		> 0.99	
	vs. *UAS*		> 0.99		0.61	
**circadian rhythm strength**		2.28 (0.17)	1.30 (0.23)	2.17 (0.19)	1.18 (0.16)	1.67 (0.20)
n		*99*	*55*	*91*	*88*	*91*
rhythmic flies (%)		82.8	38.2	72.2	28.4	52.8
significance (p) *(K-W test)*	vs. *Gal4*		< 0.001		< 0.001	
	vs. *UAS*		< 0.001		0.002	

genotype		*GMR / +*	*GMR > mAcon1^{RNAi}*	*GMR > mAcon1^{OX}*
**ERG**				
n		*12*	*10*	*11*
**max potential (mV)**		-16.58	-8.38	-11.25
significance (p) *(2way ANOVA)*	vs. *Gal4*		< 0.001	< 0.001
**max ON-transient (mV)**		3.30	0.75	2.31
significance (p) *(2way ANOVA)*	vs. *Gal4*		< 0.001	< 0.001

**max OFF-transient (mV)**		-5.84	-1.19	-5.44
significance (p)<br>*(2way ANOVA)*	vs. *Gal4*		< 0.001	< 0.001

For each genotype the mean (95% CI) or median (lifespan) and number of tested flies (n) are given. For climbing performance n refers to the number of independent experiments with 10 flies each. Activity is measured as beam breaks. While all p-values are presented, only effects that exhibit a significant difference ($p < 0.05$) from both parental controls (*Gal4* and *UAS*) are considered (red values). Statistical tests indicated: M-C, Mantel-Cox; K-W, Kruskal-Wallis.
